# Association of preoperative ankle reflex with skeletal muscle loss and poorer physical performance 6 months after gastrectomy in elderly patients with gastric cancer

**DOI:** 10.3389/fmed.2026.1873731

**Published:** 2026-08-18

**Authors:** Hailong Feng, Mingmei Xue, Lijun Meng, Linshuai Xing, Wancheng Xiong

**Affiliations:** 1 Department of Colorectal and Anal Surgery, The First Affiliated Hospital of Henan Medical University, Xinxiang, Henan, China; 2 Department of Radiology, The First Affiliated Hospital of Henan Medical University, Xinxiang, Henan, China; 3 Department of Gastroenterology, The First Affiliated Hospital of Henan Medical University, Xinxiang, Henan, China

**Keywords:** ankle reflex, elderly adults, gastric cancer, physical performance, skeletal muscle index

## Abstract

**Background:**

Neuromuscular dysfunction may contribute to postoperative skeletal muscle loss in elderly patients with gastric cancer. However, the association between preoperative ankle reflex and postoperative muscle-related outcomes remains unclear. This study investigated whether reduced or absent preoperative ankle reflexes were associated with appendicular skeletal muscle index (ASMI) and impaired physical performance 6 months after gastrectomy.

**Methods:**

A total of 507 elderly patients with gastric cancer were enrolled. Ankle reflex was assessed within 1 week before surgery and graded from 0 to 4. Reflex status was classified as reduced or absent (grades 0–1), normal (grade 2), or brisk/hyperreflexic (grades 3–4). ASMI was measured before surgery and at 6 months after surgery using bioelectrical impedance analysis. Dominant hand grip strength, 6-m walking speed, and Short Physical Performance Battery (SPPB) scores were assessed 6 months after surgery. The primary outcome was the change in ASMI between the preoperative and 6-month postoperative assessments. The secondary outcomes included ASMI, dominant-hand grip strength, 6-m walking speed, and SPPB score at 6 months after surgery. Five self-reported muscle-related symptoms during follow-up were listed as exploratory outcomes. Multivariable linear and logistic regression models were adjusted for prespecified baseline confounders, and inverse probability weighting (IPTW) was employed as a sensitivity analysis.

**Results:**

Patients with ankle reflex grades 0–1 experienced greater ASMI change during the first 6 months after surgery than patients with normal reflexes (adjusted β = 0.311, 95% CI: 0.278–0.344, *p* < 0.001). At 6 months, they also had lower ASMI (adjusted β = −0.409), lower dominant-hand grip strength (adjusted β = −3.043), slower walking speed (adjusted β = −0.105) and lower SPPB scores (adjusted β = −1.081) (all *p* < 0.001). Reduced or absent reflexes were associated with higher odds of several exploratory muscle-related symptoms (*p* < 0.05). IPTW analyses confirmed the associations with all continuous outcomes and with selected exploratory muscle-related symptoms.

**Conclusion:**

Reduced or absent preoperative ankle reflexes were associated with greater skeletal muscle loss, as well as poorer muscle strength and physical performance, 6 months after gastrectomy in elderly patients with gastric cancer.

## 1 Introduction

Gastric cancer ranks as the fifth most common malignant tumor worldwide, with over 900,000 new cases diagnosed annually in recent years, imposing a substantial global disease burden (1, 2). China is a high-incidence region, accounting for more than 450,000 new cases each year-approximately half of the global total (3, 4). Elderly individuals aged 60–80 years constitute the core high-risk group, representing over 60% of all gastric cancer patients (5). Older patients often exhibit age-related physiological decline, multiple comorbidities, nutritional vulnerability and reduced tolerance to surgical and systemic anticancer treatments. These characteristics increase their risk of postoperative functional deterioration and may adversely affect treatment completion, quality of life, and survival (5).

Sarcopenia is a progressive disorder of skeletal muscle characterized by abnormalities in muscle mass, strength, and physical performance. It is prevalent among older patients with gastric cancer and may be exacerbated by inadequate nutrition, cancer-related systemic inflammation, decreased physical activity, surgical stress, and anticancer treatment (6–10). A growing body of evidence has associated sarcopenia or low skeletal muscle mass with postoperative complications, treatment -related toxicity, reduced tolerance to treatment, and poor survival outcomes in patients with gastric cancer (11–13). Consequently, identifying patients at risk of postoperative muscle loss and functional impairment early on is clinically important. However, sarcopenia is a multidimensional condition and a postoperative reduction in ASMI alone does not necessarily constitute a formal sarcopenia diagnosis. Muscle strength and physical performance must also be considered within an established diagnostic framework.

Previous studies have reported various factors associated with sarcopenia and muscle loss in patients with gastric cancer, including older age, lower body mass index (BMI), malnutrition, reduced protein intake, systemic inflammation, advanced tumor characteristics and exposure to anticancer treatment (14–16). By contrast, clinical indicators of neuromuscular function have received relatively little attention. Ankle reflex is a simple component of the neurological examination that depends on the functional integrity of peripheral sensory and motor pathways, spinal reflex circuits, neuromuscular junctions, and the contractile response of the involved muscles. As a classic physiological indicator reflecting lower limb nerve conduction function and muscle excitability, it is simple and non-invasive to measure (17). Reduced or absent ankle reflexes may therefore reflect underlying age-related or disease-related neuromuscular impairment. Such impairment could potentially be associated with reduced muscle activation, decreased mobility, and subsequent muscle loss. Nevertheless, ankle reflex grading is a nonspecific and examiner-dependent clinical measure, and its association with postoperative muscle-related outcomes in elder patients with gastric cancer has not been adequately investigated.

Therefore, we conducted a prospective cohort study to examine whether preoperative ankle reflex status was associated with skeletal muscle loss during the first 6 months after gastrectomy, as well as with muscle strength and physical performance at the 6-month assessment, in older patients with gastric cancer. Our hypothesis was that reduced or absent preoperative ankle reflexes would be associated with greater appendicular skeletal muscle index (ASMI) loss during the first 6 months after surgery and poorer muscle-related outcomes at the 6-month assessment. The primary outcome was ASMI loss from baseline to 6 months after surgery. The secondary outcomes were ASMI, dominant-hand grip strength, 6-m walking speed and Short Physical Performance Battery (SPPB) score at 6 months. Prespecified muscle-related symptoms during follow-up were evaluated as exploratory outcomes. As muscle strength and physical performance were not systematically assessed prior to surgery, the study evaluated postoperative muscle-related outcomes rather than the incidence of formally diagnosed postoperative sarcopenia.

## 2 Materials and methods

### 2.1 Ethical requirements

This study protocol was approved by the Medical Ethics Committee of The First Affiliated Hospital of Henan University of Medicine.

### 2.2 Study population

Consecutive patients diagnosed with gastric cancer who were admitted to the Department of Gastrointestinal Surgery, The First Affiliated Hospital of Henan University of Medicine, between January 1, 2018, and December 31, 2024, were enrolled in this study if they met the following eligibility criteria.

Inclusion criteria: (1) Aged ≥ 60 years; (2) Pathologically confirmed diagnosis of gastric cancer; (3) Underwent surgical treatment (including total gastrectomy or partial gastrectomy), with no preoperative radiotherapy, chemotherapy, or other anti-tumor therapies administered; postoperative radiotherapy, chemotherapy, or other adjuvant therapies were allowed based on individual disease conditions; (4) No previous diagnosis of sarcopenia and no known primary neuromuscular disorder or other condition expected to substantially affect skeletal muscle status. Patients with ASMI below the sex-specific AWGS 2019 threshold (men < 7.0 kg/m^2^, women < 5.7 kg/m^2^) were also excluded (18); (5) No history of other malignant tumors, and no poorly controlled or acutely exacerbated chronic diseases (e.g., acute myocardial infarction); (6) Voluntarily agreed to participate in this study, including preoperative surveys and 6-month postoperative follow-up, and signed a written informed consent form.

Exclusion criteria: (1) Failure to meet any of the above inclusion criteria; (2) Incomplete clinical data related to the study, which precluded further data analysis; (3) Refusal of follow-up, loss to follow-up, tumor recurrence, development of other major diseases, or death during the follow-up period; (4) Failure to cooperate with the completion of muscle-related assessments at the end of the 6-month follow-up period.

### 2.3 Preoperative information collection

Within 1 week before surgery, researchers collected the following information by reviewing medical records and interviewing immediate family members: demographic information, personal history, and disease history. Detailed items are listed in Table 1. The definition of personal medical history is provided in the footnote of Table 1. Disease history was confirmed based on the diagnostic certificates documented in the medical records.

**TABLE 1:** Basic characteristics of the patients before surgery.

Variable^1^	Reflex reduced/absent^3^	Reflex Normal	t/χ^2^	*P*
Total (n)	186 (36.7)	321 (63.3)	-	-
Demographic information				
Age at enrollment (year)	71.6 ± 6.5	70.3 ± 5.6	2.309	0.021
Male (n)	122 (65.6)	191 (59.5)	1.849	0.174
BMI (kg/m^2^)	21.1 ± 2.7	21.8 ± 3.1	2.520	0.012
Personal history 2				
Cigarette smoking (n)	70 (37.6)	108 (33.6)	0.823	0.364
Alcohol consumption (n)	57 (30.6)	83 (25.9)	1.351	0.245
Unhealthy diet (n)				
Pickled/grilled food intake	81 (43.5)	124 (38.6)	1.183	0.277
Inadequate vegetable/fruit intake	94 (50.5)	149 (46.4)	0.801	0.371
Irregular meal patterns	98 (52.7)	156 (48.6)	0.788	0.375
High-salt diet	87 (46.8)	141 (43.9)	0.386	0.534
Insufficient physical activity (n)	125 (67.2)	178 (55.5)	6.764	0.009
Disease history				
Type 2 diabetes (n)	45 (24.2)	57 (17.8)	3.036	0.081
Hypertension (n)	86 (46.2)	135 (42.1)	0.837	0.360
Coronary heart disease (n)	53 (28.5)	79 (24.6)	0.923	0.337
Cerebrovascular disease (n)	31 (16.7)	42 (13.1)	1.226	0.268
Gastric ulcer (n)	28 (15.1)	40 (12.5)	0.682	0.409
Chronic atrophic gastritis (n)	44 (23.7)	52 (16.2)	4.266	0.039
Hp infection (n)	97 (52.2)	158 (49.2)	0.404	0.525
Muscle indicator				
ASMI (kg/m^2^)	7.5 ± 0.7	7.6 ± 0.7	1.508	0.132

^1^ASMI, Appendicular skeletal muscle mass index.

^2^Definition of personal history: Cigarette smoking: ≥ 1 cigarette consumed weekly; Alcohol consumption: ≥ 4 alcoholic drinks per week (1 drink ≈ 14 g pure alcohol); Pickled/grilled food intake: ≥ 3 servings weekly, 100 g per serving at least; Inadequate vegetable/fruit intake: Daily combined intake < 500 g for ≥ 5 days weekly; Irregular meal patterns: Irregular eating behaviors (breakfast skipping, dinner after 19:00, or inter-meal intervals > 6 h) on ≥ 4 days weekly; High-salt diet: Daily salt intake ≥ 6 g (including condiments); Insufficient physical activity: Weekly moderate-intensity exercise < 150 min or high-intensity exercise < 75 min. All above personal histories indicate a cumulative duration of ≥ 1 year within the past 10 years.

^3^Continuous variables were expressed as mean ± standard deviation. Categorical variables were expressed as frequency and constituent ratio. A value of *P* < 0.05 indicated statistical significance.

### 2.4 Preoperative assessment and examination

Appendicular skeletal muscle mass (ASM) was performed by bioelectrical impedance analysis (BIA) using the InBody 370 analyzer (Biospace Co., Ltd., Republic of Korea). Appendicular lean mass was calculated as the sum of the lean mass estimates for both arms and both legs. The appendicular skeletal muscle index was calculated by dividing this value by height squared. All measurements were performed by well-trained researchers in the ward within 1 week before surgery in accordance with the manufacturer’s standardized protocol. Specifically, participants were required to fast for at least 8 h prior to the test, emptied their bladder before assessment, removed their shoes and any metal objects, and remained standing in the standardized position specified by the manufacturer. Patients with clinically apparent edema, ascites, or who had received intravenous fluids within 48 h were reassessed after stabilization (19). Ankle reflex was tested bilaterally within 1 week before surgery using a standard neurological reflex hammer. Participants were examined in the supine position with their lower limbs relaxed. The Achilles tendon was gently tapped to elicit a plantar flexion response. Absent (grade 0), reduced (grade 1), normal (grade 2), exaggerated (grade 3) and markedly hyperreflexic with obvious clonus (grade 4) were the grades that were assigned (17, 20). The two investigators were unaware of each other’s ratings, and all ratings were recorded before any discussion or adjudication. The rating assigned by the prespecified primary examiner was used to define the exposure in the primary analyses, whereas the second examiner’s rating was used to evaluate inter-rater reliability. Asymmetry was recorded when there was a difference of ≥ 1 grade in reflex grading between the left and right sides. When the right and left ankle reflex grades differed, the lower grade was used as the participant-level exposure because the primary interest was the identification of reduced reflex function. Although the complete 0–4 grading scale was applied, only grades 0–2 was observed in this cohort. Participants were classified as having reduced or absent reflexes (grades 0–1) or normal reflexes (grade 2). Grades 3–4 were considered brisk or hyperreflexic and would not have been classified as normal had they been observed.

### 2.5 Postoperative follow-up

Three rounds of follow-up were conducted at hospital discharge after surgery, as well as at 3 and 6 months postoperatively. The follow-up protocol involved reviewing medical records and conducting home visits to interview family members to collect the following information: pathological features, surgical procedures and postoperative treatment regimens, postoperative (out-of-hospital) complications and corresponding treatments within 6 months, and postoperative (out-of-hospital) dietary patterns and physical activity levels within 6 months. Detailed indicators are listed in Tables 2–4.

**TABLE 2:** Pathological features, surgical and postoperative treatment.

Variable	Reflex reduced/absent^1^	Reflex normal	*t*/χ^2^	*P*
Total (n)	186 (36.7)	321 (63.3)	-	-
Pathological feature				
Tumor location (n)				
Cardia-gastric fundus	71 (38.2)	98 (30.5)	3.095	0.079
Gastric body/antrum	115 (61.8)	223 (69.5)		
Tumor differentiation (n)				
Well/moderately differentiated	59 (31.7)	132 (41.1)	4.433	0.035
Poorly differentiated/signet ring	127 (68.3)	189 (58.9)		
Depth of invasion (n)				
T1-T2	58 (31.2)	116 (36.1)	1.282	0.257
T3-T4	128 (68.8)	205 (63.9)		
Lymph node metastasis N1-N3 (n)	123 (66.1)	194 (60.4)	1.629	0.202
Distant metastasis M1 (n)	7 (3.8)	13 (4.0)	0.025	0.873
Vascular invasion (n)	79 (42.5)	119 (37.1)	1.444	0.230
Neural invasion (n)	68 (36.6)	105 (32.7)	0.776	0.378
Surgical information				
Surgical method (n)				
Total gastrectomy	78 (41.9)	120 (37.4)	1.025	0.311
Partial gastrectomy	108 (58.1)	201 (62.6)		
Lymph node dissection (n)				
D1 dissection	22 (11.8)	48 (15.0)	0.967	0.326
D2 and extended dissection	164 (88.2)	273 (85.0)		
Surgical approach (n)				
Laparoscopic/robotic-assisted	109 (58.6)	201 (62.6)	0.799	0.371
Open surgery	77 (41.4)	120 (37.4)		
Intraoperative blood transfusion (n)	53 (28.5)	76 (23.7)	1.441	0.230
Postoperative hospital stay (n)				
≤ 14 days	108 (58.1)	216 (67.3)	4.345	0.037
>14 days	78 (41.9)	105 (32.7)		
Postoperative adjuvant therapy				
Chemotherapy (n)	126 (67.7)	201 (62.6)	1.351	0.245
Chemotherapy cycles (n)				
≤ 4 cycles	77 (61.1)	138 (68.7)	1.958	0.162
>4 cycles	49 (38.9)	63 (31.3)		
Radiotherapy (n)	38 (20.4)	59 (18.4)	0.320	0.572
Targeted/immunotherapy (n)	22 (11.8)	39 (12.1)	0.012	0.915

^1^Categorical variables were expressed as frequency and constituent ratio. A value of *P* < 0.05 indicated statistical significance.

**TABLE 3:** Postoperative (out-of-hospital) complications and treatments within 6 months.

Postoperative complications and treatments^1^	Reflex reduced/absent^2^	Reflex normal	*t*/χ^2^	*P*
Total (n)	186 (36.7)	321 (63.3)	-	-
Postoperative gastroparesis				
Case (n)	34 (18.3)	47 (14.6)	1.161	0.281
Prokinetic agents (n)	28 (82.4)	42 (89.4)	0.826	0.364
Gastric decompression + IVN (n)	6 (17.6)	5 (10.6)		
Reflux esophagitis				
Case (n)	43 (23.1)	51 (15.9)	4.076	0.043
PPI (n)	36 (83.7)	47 (92.2)	1.607	0.205
PPI + prokinetic agents (n)	7 (16.3)	4 (7.8)		
Chronic diarrhea				
Case (n)	28 (15.1)	41 (12.8)	0.521	0.470
Intestinal flora regulators (n)	15 (53.6)	25 (61.0)	0.374	0.541
Antidiarrheal agents (n)	13 (46.4)	16 (39.0)		
Severe constipation				
Case (n)	23 (12.4)	35 (10.9)	0.249	0.618
Laxatives (n)	18 (78.3)	31 (88.6)	1.126	0.289
Enema treatment (n)	5 (21.7)	4 (11.4)		
Moderate-severe malnutrition				
Case (n)	61 (32.8)	77 (24.0)	4.612	0.032
ONS (n)	48 (78.7)	65 (84.4)	0.753	0.386
NF-EN (n)	13 (21.3)	12 (15.6)		
Hypoalbuminemia				
Case (n)	48 (25.8)	67 (20.9)	1.635	0.201
Albumin infusion (n)	14 (29.2)	22 (32.8)	0.175	0.676
High-protein diet + ONS (n)	34 (70.8)	45 (67.2)		
Electrolyte disturbance				
Case (n)	33 (17.7)	50 (15.6)	0.403	0.525
Oral electrolyte supplements (n)	23 (69.7)	41 (82.0)	1.705	0.192
IV electrolyte replacement (n)	10 (30.3)	9 (18.0)		
Pneumonia				
Case (n)	19 (10.2)	28 (8.7)	0.312	0.577
Oral antibiotics (n)	11 (57.9)	20 (71.4)	0.923	0.337
IV antibiotics (n)	8 (42.1)	8 (28.6)		

^1^IVN, Intravenous nutrition; PPI, Proton pump inhibitor; NF-EN, Nasal feeding enteral nutrition; ONS, Oral nutritional supplement; IV, Intravenous.

^2^Categorical variables were expressed as frequency and constituent ratio. A value of *P* < 0.05 indicated statistical significance.

**TABLE 4:** Postoperative (out-of-hospital) dietary patterns, and physical activities within 6 months.

Variable	Reflex reduced/absent^1^	Reflex normal	*t*/χ^2^	*P*
Total (n)	186 (36.7)	321 (63.3)	-	-
Dietary pattern				
Daily meal frequency (n)				
≤ 3 times	91 (48.9)	139 (43.3)	1.502	0.220
<3 times	95 (51.1)	182 (56.7)		
Single meal intake (n)				
<100 g	78 (41.9)	121 (37.7)	0.888	0.346
≥ 100 g	108 (58.1)	200 (62.3)		
Predominant staple food (n)				
Rice/noodles	101 (54.3)	193 (60.1)	1.639	0.200
Porridge/soup	85 (45.7)	128 (39.9)		
Eating 2 h before bed (n)				
Yes	67 (36.0)	95 (29.6)	2.237	0.135
No	119 (64.0)	226 (70.4)		
Overheated food intake (n)				
Yes	57 (30.6)	88 (27.4)	0.602	0.438
No	129 (69.4)	233 (72.6)		
Daily protein intake (n)				
<50 g/day	94 (50.5)	136 (42.4)	3.172	0.075
≥ 50 g/day	92 (49.5)	185 (57.6)		
Physical activity				
Frequency of exercise (n)				
≤ 1 time/week	108 (58.1)	154 (48.0)	4.801	0.028
≥ 2 times/week	78 (41.9)	167 (52.0)		
Single exercise duration (n)				
≤ 30 min	113 (60.8)	176 (54.8)	1.686	0.194
≥ 30 min	73 (39.2)	145 (45.2)		
Exercise type (n)				
Low-intensity (e.g., walking)	152 (81.7)	269 (83.8)	0.362	0.548
Moderate-intensity (e.g., running)	34 (18.3)	52 (16.2)		

^1^Categorical variables were expressed as frequency and constituent ratio. A value of *P* < 0.05 indicated statistical significance.

### 2.6 Postoperative muscle mass, muscle strength, and physical performance outcomes

The primary outcome was skeletal muscle loss during the first 6 months after surgery, calculated as: skeletal muscle loss = ASMI preoperative −ASMI at 6 months. A positive value therefore indicated a reduction in skeletal muscle mass.

Secondary outcomes assessed at 6 months included ASMI, dominant-hand grip strength, 6-m walking speed and SPPB score. As handgrip strength, walking speed and SPPB were not measured preoperatively, these variables were interpreted as cross-sectional outcomes at 6 months rather than as changes from the preoperative baseline.

Muscle strength assessment: Dominant hand grip strength was measured using a Jamar Hydraulic Hand Dynamometer (Patterson Medical, United States). Participants were instructed to stand upright with their arm hanging down naturally, squeeze the dynamometer maximally for 3 consecutive trials, and the highest value was recorded in kilograms (kg) (21).

Muscle function assessment: Walking speed was calculated by documenting the time taken for participants to complete a 6-m straight-line walk, with the unit expressed as meters per second (m/s) (22). Additionally, muscle function was evaluated using the Short Physical Performance Battery (SPPB). This consists of three components: standing balance, gait speed and the chair stand test. The total score ranges from 0 to 12. A higher score indicates better muscle function, whereas a lower score suggests impaired muscle function (23).

Meanwhile, the occurrence of 5 symptoms during the follow-up period was recorded, including muscle weakness, gait instability, fatigue during mild activity, difficulty in lifting light objects, and reduced standing balance. Symptoms were considered present if they were reported as occurring at least weekly at both the 3- and 6-month follow-up assessments, and the specific definitions of these symptoms are provided in the footnote of Table 5. These symptoms were treated as exploratory outcomes.

**TABLE 5:** Muscle-related outcomes at 6 months after surgery.

Variable^1^	Reflex reduced/absent^3^	Reflex normal	*t*/χ^2^	P
Total (n)	186 (36.7)	321 (63.3)	-	-
Muscle mass				
ASMI (kg/m^2^)	7.1 ± 0.8	7.5 ± 0.7	5.872	<0.001
ASMI change vs. preoperative (kg/m^2^)	0.5 ± 0.3	0.1 ± 0.1	19.263	<0.001
Muscle strength				
Dominant hand grip strength (kg)	18.7 ± 3.8	21.7 ± 4.1	8.152	<0.001
Muscle function				
6-m walking speed (m/s)	0.9 ± 0.1	1.0 ± 0.1	12.360	<0.001
SPPB score	8.0 ± 0.7	9.1 ± 1.1	11.512	<0.001
Key symptom 2				
Muscle weakness (n)	92 (49.5)	115 (35.8)	9.065	0.003
Gait instability (n)	78 (41.9)	93 (29.0)	8.854	0.003
Fatigue with mild activity (n)	103 (55.4)	138 (43.0)	7.244	0.007
Difficulty in lifting light objects (n)	86 (46.2)	107 (33.3)	8.316	0.004
Reduced standing balance (n)	72 (38.7)	88 (27.4)	6.956	0.008

^1^ASMI, Appendicular Skeletal muscle mass index; SPPB, Short physical performance battery score.

^2^Definition of key symptom: Muscle weakness: Persistent limb fatigue affecting daily activities excluding other secondary factors; Gait instability: Unsteady walking or tendency to fall without cerebrovascular or bone and joint diseases; Fatigue with mild activity: Excessive tiredness after mild exercise that cannot be relieved by short rest; Difficulty in lifting light objects: Inability to lift items ≥ 5 kg independently due to upper limb muscle weakness; Reduced standing balance: Poor balance when standing without support excluding vestibular or neurological disorders. Only symptoms that occurred on a weekly basis throughout the period were included in the statistical analysis.

^3^Continuous variables were expressed as mean ± SD. Categorical variables were expressed as frequency and constituent ratio. A value of *P* < 0.05 indicated statistical significance.

### 2.7 Statistical analysis

Continuous variables were summarized as mean ± standard deviation or the median with the interquartile range, depending on their distribution. Categorical variables were summarized as a number and percentage. Between-group comparisons were considered descriptive only and were not used to select covariates for multivariable models.

Covariates were prespecified based on previous evidence, clinical relevance and assumed causal relationships between ankle reflex status, baseline health status, treatment characteristics and postoperative muscle-related outcomes. The primary adjustment set included the following: age; sex; BMI; preoperative ASMI; type 2 diabetes; preoperative physical activity; major cardiovascular or cerebrovascular disease; tumor stage; and tumor differentiation. Postoperative malnutrition, postoperative exercise frequency, reflux esophagitis and length of hospital stay were excluded from the primary model as they occurred after ankle reflex assessment and may represent mediators rather than baseline confounders.

Multivariable linear regression was used to analyze continuous outcomes, including skeletal muscle loss, ASMI at 6 months, handgrip strength, walking speed and SPPB score. Results were reported as adjusted β coefficients with 95% confidence intervals. Multivariable logistic regression was used for binary outcomes, with the results reported as adjusted odds ratios and 95% confidence intervals.

The reflex normal group (grade 2) was used as the reference group. Reflex status was prespecified as reduced or absent (grades 0–1), normal (grade 2), and brisk or hyperreflexic (grades 3–4). However, because no participant had a grade 3 or 4 reflex, the analyses compared grades 0–1 with grade 2. Inter-rater reliability was assessed using the independent ratings of the two examiners for all 507 participants. Agreement for the original ordinal grades was assessed using linearly weighted Cohen’s kappa, whereas agreement for the binary classification [reduced or absent reflexes (grades 0–1) versus normal reflexes (grade 2)] was assessed using unweighted Cohen’s kappa. The exact agreement percentages and kappa coefficients, along with their 95% confidence intervals, were reported.

Inverse probability of treatment weighting (IPTW) was used to balance baseline characteristics between patients with normal and reduced/absent preoperative ankle reflexes. Propensity scores were estimated via logistic regression incorporating 29 covariates, including demographics (age, sex, BMI), personal history, comorbidities, tumor pathological features, and surgical characteristics. Stabilized weights were applied to estimate the average treatment effect (ATE). Covariate balance was evaluated using standardized mean differences (SMD < 0.1). Weight distribution, coefficient of variation, and effective sample size (ESS) were reported. Sensitivity analysis involved truncating weights at the 1st and 99th percentiles. A doubly robust analysis combining IPTW with covariate adjustment in the outcome model was performed as an additional sensitivity analysis. IPTW-weighted linear regression was used for continuous outcomes, and IPTW-weighted logistic regression was used for binary outcomes. Models were fitted using the survey package with model-robust standard errors. A doubly robust sensitivity analysis was also performed by combining stabilized IPTW with adjustment for the 29 covariates included in the propensity-score model.

Two-sided *P*-values below 0.05 were considered statistically significant. Interpretation emphasized effect estimates and 95% confidence intervals rather than the magnitude of individual *P*-values. All the above analyses were performed using SPSS 29.0 software (IBM Corp., Armonk, NY, United States) and R software (version 4.3.3; R Foundation for Statistical Computing, Vienna, Austria).

## 3 Results

### 3.1 Study population

Initially, a total of 528 patients who met the inclusion criteria were enrolled in this study. During the follow-up period, 12 patients were excluded due to incomplete clinical data, 5 due to refusal to cooperate with muscle-related assessments, and 4 due to loss to follow-up, no cases were excluded due to relapse, death, or serious illness. Consequently, 507 patients (96.0%) were included in the final analysis. Based on the prespecified primary examiner’s ratings, 37 patients had grade 0 reflexes, 149 had grade 1 reflexes, and 321 had grade 2 reflexes. No participant had a grade 3 or 4 reflex. Participants were therefore classified into the reduced/absent-reflex group (grades 0–1, *n* = 186) and the normal-reflex group (grade 2, *n* = 321).

### 3.2 Inter-rater reliability of ankle reflex assessment

Independent ankle reflex ratings from both examiners were available for all 507 participants. The examiners assigned the same ordinal grade to 483 participants, corresponding to exact agreement in 95.3% of cases. The linearly weighted Cohen’s kappa for grades 0–2 was 0.921 (95% CI: 0.892–0.950), indicating high inter-rater agreement. All 24 disagreements occurred between grades 0 and 1, with none involving grade 2. Consequently, the two examiners agreed on the binary classification of all participants as having either reduced or absent reflexes (grades 0–1) or normal reflexes (grade 2). Binary agreement was therefore 100%, with an unweighted Cohen’s kappa of 1.000. None of the disagreements regarding ordinal grades affected the exposure classification used in the primary analyses (see Supplementary Tables 1, 2).

### 3.3 Basic characteristics of the patients before surgery

As shown in Table 1, compared with the reflex normal group, patients in the Reflex reduced/absent Group were significantly older, had a lower BMI, presented with a higher proportion of insufficient physical activity, and had a higher prevalence of chronic atrophic gastritis (all *P* < 0.05). In addition, the proportion of patients with type 2 diabetes was also higher in the Reflex reduced/absent Group, but the difference was not statistically significant (*P* > 0.05).

### 3.4 Pathological features, surgical and postoperative treatment

As presented in Table 2, compared with the reflex normal group, patients in the reflex reduced/absent Group had a significantly higher proportion of poorly differentiated/signet ring cell carcinoma, along with a higher rate of postoperative hospital stay exceeding 14 days (all *P* < 0.05). In addition, the proportion of cardia-gastric fundus gastric cancer was higher in the Reflex reduced/absent Group, but this difference was not statistically significant (*P* > 0.05).

### 3.5 Postoperative (out-of-hospital) complications and treatments within 6 months

As illustrated in Table 3, compared with the reflex normal group, patients in the reflex reduced/absent Group exhibited a significantly higher prevalence of reflux esophagitis and moderate-to-severe malnutrition (*P* < 0.05).

### 3.6 Postoperative (out-of-hospital) dietary patterns, and physical activities within 6 months

As demonstrated in Table 4, compared with the reflex normal group, patients in the reflex reduced/absent group had a significantly higher proportion of exercise frequency ≤ 1 time per week (*P* < 0.05). The proportion of patients with daily protein intake < 50 g/day was also higher in the reflex reduced/absent group, but the difference was not statistically significant (*P* > 0.05).

### 3.7 Muscle-related outcomes at 6 months after surgery

As presented in Table 5, compared with patients with normal reflex, those with reduced or absent reflexes had lower ASMI, handgrip strength, walking speed, and SPPB scores at 6 months, and experienced greater skeletal muscle loss during follow-up (all *P* < 0.001). In addition, the proportions of patients with muscle weakness, gait instability, fatigue following mild activity, difficulty lifting light objects, and impaired standing balance during the follow-up were significantly higher in the Reflex reduced/absent Group (all *P* < 0.05).

### 3.8 Association of preoperative ankle reflex with skeletal muscle loss and impaired physical performance after gastric cancer resection

**FIGURE 1:**
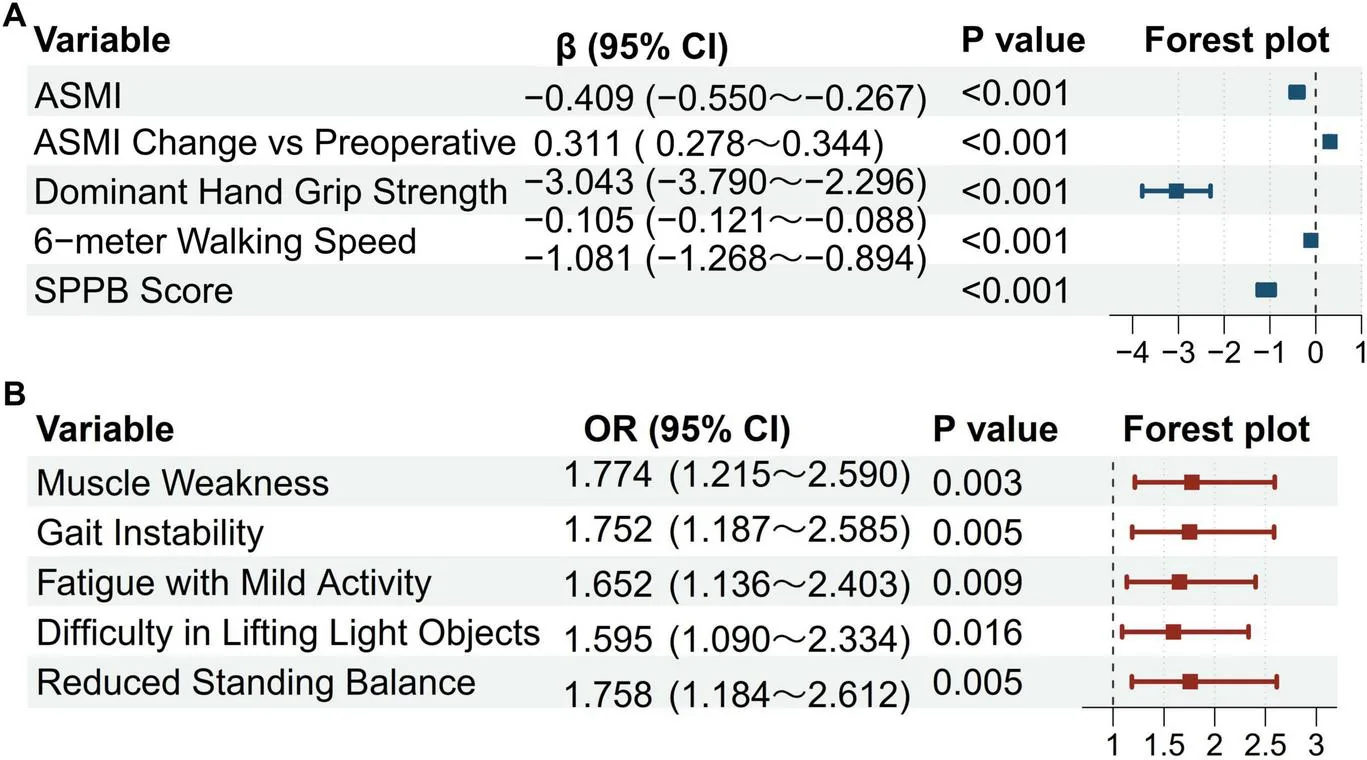
Association of preoperative ankle reflex with skeletal muscle loss and impaired physical performance after gastric cancer resection. **(A)** ASMI, Appendicular skeletal muscle mass index; SPPB, Short physical performance battery score; OR, Odds ratio; 95%CI, 95% Confidence interval. **(B)** Multivariable models were adjusted for prespecified covariates selected according to previous evidence, clinical relevance, and the assumed causal structure. These covariates included age, sex, BMI, preoperative ASMI, type 2 diabetes, preoperative physical activity, cardiovascular or cerebrovascular disease, tumor stage, and tumor differentiation.

The results of multivariate linear regression analysis for continuous variables showed that, compared with the reflex normal group, the reflex reduced/absent group had a significantly lower ASMI and a greater change in ASMI from preoperative to follow-up. Regarding muscle function, reflex reduced/absent group had significantly lower dominant-hand grip strength, 6-m walking speed, and SPPB scores than the reflex normal group, with all differences being statistically significant (all *P* < 0.001) (see Figure 1A). Results of multivariate logistic regression analysis for binary variables showed that reflex reduced/absent group had a significantly higher risk of muscle weakness, gait instability, mild post-exercise fatigue, difficulty lifting light objects, and impaired standing balance compared to the reflex normal group; all these differences were statistically significant (all *P* < 0.05) (Figure 1B).

### 3.9 Robustness analysis

Stabilized IPTW weights were estimated using 29 measured preoperative and perioperative covariates, including preoperative ASMI. Before weighting, an absolute standardized mean difference above 0.10 was observed for several covariates, including age, BMI, sex, alcohol consumption, insufficient physical activity, type 2 diabetes, chronic atrophic gastritis, preoperative ASMI, tumor location, tumor differentiation, depth of invasion, lymph-node metastasis, vascular invasion, and intraoperative blood transfusion. After stabilized IPTW, covariate balance was substantially improved. Twenty-eight of the 29 measured covariates achieved an absolute standardized mean difference below 0.10. A small residual imbalance remained for hypertension (absolute SMD = 0.102; Table 6 and Supplementary Figure 1).

**TABLE 6:** Covariate balance before and after stabilized inverse probability of treatment weighting.

Variable	Absolute SMD before IPTW^ 1^	Absolute SMD after IPTW	Absolute SMD after weight truncation
Age at enrollment	0.208	0.067	0.010
BMI	−0.237	0.065	0.010
Male	0.126	0.082	0.014
Cigarette smoking	0.083	0.028	0.007
Alcohol consumption	0.107	0.014	0.017
Pickled/grilled food intake	0.100	0.007	0.021
Inadequate vegetable/fruit intake	0.083	0.059	0.011
Irregular meal patterns	0.082	0.057	0.002
High-salt diet	0.057	0.058	0.010
Insufficient physical activity	0.243	0.036	0.031
Type 2 diabetes	0.159	0.029	0.004
Hypertension	0.84	0.102	0.057
Coronary heart disease	0.088	0.054	0.023
Cerebrovascular disease	0.101	0.015	0.008
Gastric ulcer	0.075	0.042	0.021
Chronic atrophic gastritis	0.187	0.021	0.006
Hp infection	0.059	0.064	0.013
Preoperative ASMI	0.140	0.010	0.024
Tumor location	0.161	0.033	0.003
Tumor differentiation	0.196	0.019	0.020
Depth of invasion	0.105	0.046	0.008
Lymph node metastasis N1-N3	0.118	0.024	0.018
Distant metastasis M1	0.015	0.051	0.027
Vascular invasion	0.111	0.024	0.018
Neural invasion	0.081	0.025	0.001
Surgical method	0.093	0.034	0.007
Lymph node dissection	0.092	0.023	0.026
Surgical approach	0.040	0.011	0.023
Intraoperative blood transfusion	0.110	0.022	0.004

^1^SMD, standardized mean difference; IPTW, inverse probability of treatment weighting Values represent absolute standardized mean differences calculated using standardized differences for both continuous and binary covariates. An absolute SMD < 0.10 was considered indicative of acceptable balance. After stabilized IPTW, 28 of the 29 measured covariates met the prespecified balance criterion; hypertension showed a small residual imbalance (absolute SMD = 0.102). After truncation of stabilized weights at the 1st and 99th percentiles, all 29 covariates achieved an absolute SMD below 0.10.

The stabilized weights ranged from 0.442 to 11.383, with an overall mean of 1.013 and a standard deviation of 0.611. The effective sample size decreased from 186 to 104.52 in the reduced/absent-reflex group, corresponding to a 43.8% reduction, and from 321 to 292.93 in the normal-reflex group, corresponding to an 8.7% reduction. This greater reduction in the reduced/absent-reflex group was consistent with its higher weight variability and maximum stabilized weight. After truncation at the 1st and 99th percentiles, the effective sample size in the reduced/absent-reflex group increased to 148.89, reducing the ESS loss to 20.0%, whereas the continuous-outcome estimates remained materially unchanged. After weight truncation, all 29 covariates achieved an absolute standardized mean difference below 0.10, with a maximum value of 0.057 for hypertension (Tables 6, 7).

**TABLE 7:** Distribution of stabilized IPTW weights and effective sample size.

Measure^1^	Reflex reduced/absent group	Reflex normal group
Unweighted sample size	186	321
Minimum weight	0.442	0.670
25th percentile	0.640	0.793
Median	0.846	0.901
Mean	1.040	0.994
75th percentile	1.180	1.100
Maximum weight	11.383	2.935
Coefficient of variation	0.885	0.310
Unweighted ESS	186	321
IPTW-weighted ESS	104.52	292.93
ESS reduction, %	43.8	8.7
ESS after weight truncation	148.89	293.37
ESS reduction after weight truncation, %	20.0	8.6

^1^IPTW, inverse probability of treatment weighting; ESS, effective sample size.

In the IPTW-weighted analyses, reduced or absent ankle reflexes were associated with lower ASMI at 6 months (β = −0.324 kg/m^2^, 95% *CI:*−0.462 to −0.186), greater ASMI loss from baseline to 6 months (β = 0.317 kg/m^2^, 95% *CI:* 0.278–0.356), lower dominant-hand grip strength (β = −3.766 kg, 95% *CI* : −4.623 to −2.909), slower walking speed (β = −0.107 m/s, 95% *CI* : −0.125 to −0.088), and lower SPPB scores (β = −1.051, 95% *CI:*−1.218 to −0.885; all *P* < 0.001).

For the exploratory binary outcomes, reduced or absent reflexes were associated with muscle weakness (*OR* = 1.573, 95% *CI*: 1.006–2.460) and difficulty lifting light objects (*OR* = 1.581, 95% *CI*: 1.010–2.474) in the untruncated IPTW analysis. The confidence intervals for gait instability, fatigue after mild activity, and reduced standing balance included the null value. All continuous-outcome estimates remained similar in the doubly robust and truncated-weight analyses. The binary symptom outcomes were less consistent across the analytical specifications (Table 8).

**TABLE 8:** IPTW-weighted regression analysis of preoperative ankle reflex and postoperative outcomes (6 months).

Outcome	IPTW-weighted β/OR (95%CI)^1^	Doubly robust (IPTW + covariate adjustment) β/OR (95%CI)^2^	Truncated-weight sensitivity β/OR (95%CI)^3^
Continuous outcomes			
ASMI at 6 months	−0.324(−0.462, −0.186) ***	−0.317(−0.353, −0.281) ***	−0.335 (−0.475, −0.196)***
ASMI change vs. preoperative	0.317 (0.278, 0.356) ***	0.317 (0.281, 0.353) ***	0.318 (0.279, 0.358)***
Dominant hand grip strength	−3.766 (−4.623, −2.909) ***	−3.461 (−3.755, −3.166) ***	−3.564 (−4.351, −2.778)***
6-m walking speed	−0.107 (−0.125, −0.088) ***	−0.107 (−0.124, −0.090) ***	−0.106 (−0.123, −0.088)***
SPPB score	−1.051 (−1.218, −0.885) ***	−1.061 (−1.221, −0.901) *	−1.049 (−1.219, −0.879)***
Binary outcomes (OR)			
Muscle weakness	1.573 (1.006, 2.460) *	1.683 (1.111, 2.549) *	1.695 (1.134, 2.532) *
Gait instability	1.420 (0.913, 2.210)	1.490 (0.974, 2.278)	1.552 (1.030, 2.338) *
Fatigue with mild activity	1.332 (0.851, 2.084)	1.484 (0.984, 2.241)	1.493 (1.002, 2.222) *
Difficulty lifting light objects	1.581 (1.010, 2.474) *	1.731 (1.135, 2.641) *	1.690 (1.127, 2.534) *
Reduced standing balance	1.548 (0.986, 2.432)	1.630 (1.063, 2.501) *	1.673 (1.100, 2.544) *

^1^β, regression coefficient (Reduced/absent vs. Normal group); OR, odds ratio.

^2^IPTW, inverse probability of treatment weighting (stabilized weights, ATE estimand). Doubly robust model additionally adjusted for all 29 measured preoperative and perioperative covariates listed in Table 6.

^3^Truncated-weight sensitivity analysis truncated weights at the 1st/99th percentiles. The null value was 0 for β coefficients and 1 for Ors.

*P < 0.05, **P < 0.01, ***P < 0.001.

## 4 Discussion

To our knowledge, this is one of the first prospective cohort studies to examine the link between a common neurological test performed at the patient’s bedside and postoperative muscle-related outcomes in older patients undergoing gastrectomy for gastric cancer. Reduced or absent preoperative ankle reflexes were associated with greater appendicular skeletal muscle index (ASMI) loss during the first 6 months after surgery, as well as poorer objective muscle-related outcomes at the 6-month assessment. These findings build on previous research which focused primarily on nutritional, inflammatory and oncological factors, and suggest that routine neuromuscular examination could offer further clinical insights into postoperative muscle deterioration. However, the observed associations should not be interpreted as evidence that reduced ankle reflexes directly cause skeletal muscle loss or functional impairment.

This study focuses specifically on elderly individuals because gastric cancer predominantly affects this population, and because aging is accompanied by a progressive vulnerability to skeletal muscle loss, reduced muscle strength and impaired physical performance. Elderly patients with gastric cancer may experience reduced physiological reserves, poorer tolerance of treatment, nutritional deterioration and slower postoperative recovery than younger patients (5). These muscle-related impairments are clinically important as they negatively impact functional independence, treatment completion, quality of life and prognosis. While these outcomes are key components of sarcopenia, the present study did not formally diagnose sarcopenia according to consensus criteria. Therefore, our findings are interpreted in terms of postoperative skeletal muscle loss and muscle-related functional outcomes rather than incident postoperative sarcopenia.

The observed associations involved complementary dimensions of muscle health. ASMI was measured before surgery and again at 6 months, enabling the longitudinal evaluation of skeletal muscle loss. Handgrip strength, walking speed and SPPB scores, meanwhile, provided objective information on muscle strength and physical performance at the 6-month assessment. These objective measures yielded broadly consistent findings, supporting a multidimensional association between reduced or absent ankle reflexes and adverse postoperative muscle-related outcomes. Muscle-related symptoms were also more prevalent in the reduced/absent-reflex group in the primary analysis. However, these exploratory symptom outcomes were less consistent across the weighted sensitivity analyses and should therefore be interpreted with caution. Furthermore, as handgrip strength, walking speed and SPPB scores were not assessed preoperatively, the study was unable to determine whether the observed between-group differences at 6 months represented greater postoperative deterioration or pre-existing functional differences. Longitudinal change could only be evaluated for ASMI, and the exploratory symptoms were not used as diagnostic criteria for sarcopenia.

A key strength of this study was the repeated assessment of skeletal muscle mass, both before surgery and at 6-month follow-up. In the unadjusted comparison, preoperative ASMI was similar between the reflex groups, whereas patients with reduced or absent reflexes subsequently experienced greater skeletal muscle loss. This longitudinal assessment mitigates, though does not eliminate, concerns that the postoperative difference in muscle mass was entirely attributable to baseline ASMI. Preoperative ASMI was therefore included as a covariate in the adjusted analysis. Independent ankle reflex assessments by two trained examiners showed high inter-rater agreement. All disagreements occurred between grades 0 and 1 and therefore did not affect the binary exposure classification used in the primary analyses. Nevertheless, this agreement reflects reproducibility under the standardized conditions of a single-center study rather than diagnostic validity or external reproducibility.

However, the original eligibility criterion referred to the absence of a documented history of sarcopenia, rather than excluding sarcopenia through comprehensive, standardized assessment. All participants had a preoperative ASMI above the sex-specific AWGS 2019 thresholds. Therefore, none met the low-muscle-mass criterion before surgery. However, because handgrip strength and physical performance were not systematically assessed preoperatively, baseline low muscle strength, poor physical performance, and possible sarcopenia could not be evaluated. Consequently, the study cannot determine whether all functional abnormalities observed at 6 months developed after surgery. Instead, the results should be interpreted as associations between preoperative ankle reflex status and skeletal muscle loss or muscle-related outcomes observed during the first 6 months after surgery.

IPTW was used in a complementary sensitivity analysis to improve comparability of the measured characteristics between the reflex groups. Following stabilized weighting, 28 of the 29 measured covariates met the pre-specified balance criterion, though a slight residual imbalance remained for hypertension. The maximum stabilized weight was 11.383 and the effective sample size in the reduced/absent reflex group decreased from 186 to 104.52 indicating that a small number of observations had a significant impact on the weighted analysis. After truncating the weights at the 1st and 99th percentiles, all measured covariates achieved an absolute standardized mean difference below 0.10 and the effective sample size in the reduced/absent reflex group increased to 148.89. The associations with ASMI loss, ASMI at 6 months, grip strength, walking speed and SPPB scores remained consistent across untruncated IPTW, doubly robust and truncated-weight analyses. In contrast, the exploratory symptom outcomes were less consistent across analytical specifications. These results provide stronger support for the continuous objective outcomes than for the exploratory symptom outcomes. However, it should be noted that the weighted analyses only account for measured and appropriately modeled covariates, and cannot eliminate residual or unmeasured confounding.

There are several potential mechanisms that could explain the observed association. The ankle reflex is a spinal stretch reflex involving muscle spindle afferents, spinal integration, alpha motor neurons, peripheral motor axons, neuromuscular transmission and the contractile response of the triceps surae muscles (24). Reduced or absent ankle reflexes may therefore serve as a non-specific clinical indicator of alterations in one or more components of this reflex pathway. However, ankle reflexes may diminish with normal aging and should not be interpreted in isolation as evidence of a specific neurological disorder (25). Age-related alterations in motor neurons, motor units, neuromuscular junctions and muscle fibers have been associated with impaired neuromuscular activation and poorer motor performance (26, 27). These changes may coexist with reduced mobility and physical inactivity, leading to lower muscle contractile activity and reduced mechanical loading, which may contribute to skeletal muscle atrophy (28). Therefore, the association observed in the present study may reflect shared neuromuscular and activity-related processes associated with aging, rather than reduced ankle reflexes directly causing skeletal muscle loss.

The observed association may also be due to shared clinical and pathophysiological factors. Older patients with gastric cancer often experience systemic inflammation, poor nutrition, metabolic abnormalities and reduced physical activity. These factors can negatively impact peripheral neuromuscular function and promote skeletal muscle catabolism, impaired muscle protein synthesis and reduced physical performance (29, 30). Diabetes, nutritional deterioration and general disease severity are also associated with diminished ankle reflexes and postoperative muscle loss. Therefore, reduced ankle reflexes may partly function as a marker of broader physiological vulnerability rather than as an independent biological determinant of muscle deterioration. Although measured confounders were included in the adjusted analyses, residual confounding by inflammatory, nutritional, neurological and metabolic factors cannot be excluded.

There is also a biologically plausible bidirectional relationship between neuromuscular impairment and skeletal muscle deterioration. Reduced neuromuscular activation results in decreased muscle use and mechanical loading. Conversely, loss of muscle mass and strength can restrict mobility and neuromuscular stimulation further (31, 32). This interaction may explain why older adults often experience diminished ankle reflexes, muscle loss and impaired physical performance at the same time. However, the present study did not include nerve conduction studies, electromyography, quantitative reflex testing, analysis of inflammatory biomarkers, or muscle metabolic measurements. Thus, the proposed mechanisms remain hypothetical and require direct investigation in future studies.

This study also has several limitations. First, handgrip strength and physical performance were not assessed preoperatively; therefore, longitudinal changes could be evaluated only for ASMI. Second, BIA-derived muscle estimates may be affected by hydration and nutritional status. Although inter-rater agreement for ankle reflex classification was high, the assessment remained examiner dependent, and intra-rater and external reproducibility were not evaluated. Third, despite multivariable adjustment and IPTW analyses, residual confounding from unmeasured neurological, inflammatory, nutritional, and treatment-related factors remains possible. Selection bias may also have arisen because the analysis was restricted to patients who completed the 6-month assessment, and excluding patients with low preoperative ASMI may limit generalizability. Finally, detailed chemotherapy information was limited, and the 6-month follow-up period was insufficient to evaluate long-term outcomes.

In conclusion, elder patients with gastric cancer who had reduced or absent preoperative ankle reflexes experienced greater skeletal muscle loss during the first 6 months after surgery, as well as lower muscle strength and poorer physical performance at 6 months. These findings are preliminary and demonstrate an association rather than a causal or independently validated predictive relationship. However, ankle reflex examination could provide Supplementary Information at the bedside for identifying patients who require comprehensive nutritional, muscle and functional assessments. Future prospective studies should incorporate standardized preoperative and postoperative assessments, objective neurological testing, inflammatory and metabolic biomarkers, detailed treatment information and longer follow-up periods in order to validate the clinical relevance of these findings.

## Data Availability

The original contributions presented in the study are included in the article/Supplementary material, further inquiries can be directed to the corresponding author.
