# Supplementary material for: Association of preoperative ankle reflex with skeletal muscle loss and poorer physical performance 6 months after gastrectomy in elderly patients with gastric cancer
Source: Front Med (Lausanne). 2026 Aug 18;13:1873731. doi: 10.3389/fmed.2026.1873731 (PMC13529652; doi:10.3389/fmed.2026.1873731)
Supplement: Supplementary file 1 [file Supplementary_file_1.docx]

**Supplementary Table S1 Inter-rater reliability of preoperative ankle reflex assessment**

| **Assessment** | **Participants**  **(n)** | **Exact agreement**  **n (%)** | **Cohen’s κ** | **95% CI** |
| --- | --- | --- | --- | --- |
| Original ordinal grades, 0–2 | 507 | 483(95.3) | 0.921 ^1^ | 0.892–0.950 |
| Binary classification, grades 0–1 vs grade 2 | 507 | 507(100) | 1.000 ^2^ | Not estimable ^3^ |

¹ Linearly weighted Cohen’s κ was used because the original ankle reflex grades were ordinal. ² Unweighted Cohen’s κ was used for the binary classification. ³ The 95% confidence interval for binary Cohen’s kappa was not estimated because no discordant classifications were observed.

**Supplementary Table S2 Cross-tabulation of preoperative ankle reflex grades assigned by the two examiners**

| **Examiner 1 rating** | **Examiner 2: Grade 0** | **Examiner 2: Grade 1** | **Examiner 2: Grade 2** | **Total** |
| --- | --- | --- | --- | --- |
| Grade 0 | 24 | 13 | 0 | 37 |
| Grade 1 | 11 | 138 | 0 | 149 |
| Grade 2 | 0 | 0 | 321 | 321 |
| Total | 35 | 151 | 321 | 507 |

**Supplementary Figure S2** Covariate Balance Before and After IPTW
